# *OsMAPKKK69* Negatively Regulates Resistance to Blast and Bacterial Blight Diseases in Rice (*Oryza sativa* L.)

**DOI:** 10.3390/plants14162566

**Published:** 2025-08-18

**Authors:** Dewei Yang, Yidan Jin, Niqing He, Shaojun Lin, Zhaoping Cheng, Fenghuang Huang, Haifeng Zhang, Qingshun Q. Li, Wenquan Yu

**Affiliations:** 1 Institute of Rice, Fujian Academy of Agricultural Sciences, Fuzhou 350018, China; 2 Institute of Resources, Environment and Soil Fertilizer, Fujian Academy of Agricultural Sciences, Fuzhou 350003, China; 3 Biomedical Sciences, College of Dental Medicine, Western University of Health Sciences, Pomona, CA 91766, USA; 4 Tea Research Institute, Fujian Academy of Agricultural Sciences, Fuzhou 350013, China

**Keywords:** rice, blast disease, blight disease, *OsMAPKKK69*, disease response

## Abstract

Rice blast is one of the main diseases of rice, causing severe economic losses to agricultural production; thus, the search for blast resistance is a top priority for rice breeding. When challenged by the blast causal fungus *Magnaporthe oryzae* the expression level of *OsMAPKKK69* gene in rice cultivar Nipponbar was found to increase significantly. Such an induction was also found in a different genetic material, cultivar Shufanggaonuo, indicating that *OsMAPKKK69* plays an important role in blast disease response. However, the function of *OsMAPKKK69* remains unclear. In this study, wild type ZH11 was selected as the background material to investigate the expression and functions of *OsMAPKKK69* in rice disease resistance by constructing knockout mutants. The results showed that *OsMAPKKK69* is mainly expressed in *four-week-old shoots* and localized in cell membrane, cytoplasm, and nucleus. The two allelic knockout mutants, *osmapkkk69-1* and *osmapkkk69-2*, were more resistant to *M. oryzae* and bacterial blight *Xanthomonas oryzae* pv. *Oryzae* (*Xoo*). Further agronomic trait analysis revealed that the *osmapkkk69-1* and *osmapkkk69-2* mutants had reduced plant height, smaller grain size, a significant increase in tillering number, but also a significant increase in yield per plant. Our results show that *OsMAPKKK69* is involved in the immune response of rice by negatively regulating the resistance to rice blast and blight diseases, and in regulating important agronomic traits. This study lays a foundation for revealing the molecular mechanism of *OsMAPKKK69* in the immune response to rice diseases and provides novel genetic resources for rice breeding.

## 1. Introduction

Rice is one of the world’s major food crops, but in recent years, it has increasingly been threatened by rice blast, a globally prevalent disease caused by the rice blast fungus *Magnaporthe oryzae* [1]. Rice blast not only causes a significant reduction in yield (10% to 35%, in severe cases) but can also lead to total crop failure [2,3]. To reduce the harm of rice blast, researchers cloned nearly 30 rice blast resistance genes from different rice germplasm resource materials, including *Pit*, *Pi2*, *Pi9*, *pizt*, *Pigm*, *Pigm-1*, *Pib*, and *Pita* [4,5]. These *R* genes play an important role in the breeding of rice resistant to rice blast and they typically encode NLR (nucleotide-binding leucine-rich repeat receptors) proteins that mediate the immune defense system, leading to ETI (effector trigger immunity) response to pathogen effectors [6].

In addition to the defense system triggered by the ETI response, plants have another defense system, namely the PTI response (PAMP-triggered immunity), which is achieved through pattern recognition receptors (PRRs) on the cell surface. PRRs recognize the conserved pathogen-associated molecular patterns or microbial-associated components (PAMPs/MAMPs), thereby triggering a plant immune response [4,6]. Compared with ETI, the PTI response of plants has better broader spectrum and greater persistence [7,8].

Researchers have identified over 70 regulatory factors related to rice resistance by using different methods [2] where mitogen-activated protein kinase kinase kinase (MAPKKK)-related genes are involved in the immune response of plants [9,10,11]. Recent studies have found that the *OsMAPKKK*-related genes in rice are involved in the immune response. For instance, studies have found that *OsEDR1* (*OsMAPKKK1*) regulates the resistance of *rice bacterial blight* [12]; *OsRbg1* (*OsMAPKKK67*) negatively regulates abscisic acid (ABA) signaling and positively regulates resistance to *Burkholderia glumae* [13]. However, *OsILA1* (*OsMAPKKK43*) negatively regulates the resistance to rice bacterial blight [14]. Overexpression of *OsMAPKKKε* (*OsMAPKKK24*) increases chitin-induced *MAPK3/6* activation, while knockdown of *OsMAPKKKε* weakens chitin-induced *MAPK3/6* activation and resistance to rice blast [15]. Both *OsMAPKKK11* and *OsMAPKKK18* can regulate chitin-induced immune responses in rice, and silencing either of them reduces the activation of chitin-induced MAPK [16].

Through transcriptome analysis, we previously found that the expression of the *OsMAPKKK69* gene was significantly increased upon initial inoculation of the susceptible variety Nipponbare (Nip) Sunny with the rice blast fungus *M. oryzae* (Guy11) [17]. However, it is not clear whether the *OsMAPKKK69* gene is involved in the immune response of rice. In this study, wild type ZH11 was selected as the background material and used to construct knockout mutants of the *OsMAPKKK69* gene to explore the function of this gene in rice disease resistance.

## 2. Results

### 2.1. Transcript Accumulation of *OsMAPKKK69* in Response to M. oryzae Infection

**Figure 1:**
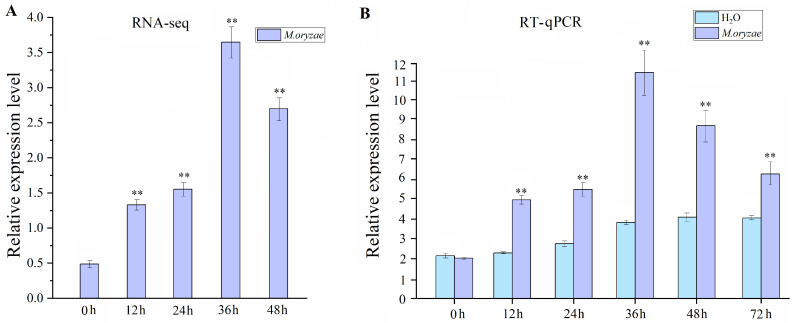
The expression analysis of *OsMAPKKK69* after *M. oryzae* infection. (**A**) Transcriptome sequencing analysis of the rice Nip samples at 0, 12, 24, 36, and 48 h after *M. oryzae* infection showed that the expression level of *OsMAPKKK69* was induced by *M. oryzae* (Guy11) infection. (**B**) RT-qPCR analysis of Shufanggaonuo rice plants at 0, 12, 24, 36, 48, and 72 h after *M. oryzae* infection revealed that the expression level of *OsMAPKKK69* was elevated and peaked at 36 *hpi*. The data analysis was conducted using Student’s *t*-test; ** *p* ≤ 0.01.

Previously [17], the wild type genotype Nip was infected with isolates from the blast fungus Guy11, and transcriptome sequencing and analysis were performed at different time points of 0, 12, 24, and 48 h after inoculation. The results showed that the expression of the *LOC_Os05g46760* gene was significantly elevated in response to Guy11 (Figure 1A), and according to the previous studies [18], this gene was named *OsMAPKKK69*. To further verify the expression of *OsMAPKKK69* after induction by Guy11 in different genetic materials, we used the collected germplasm resource Shufanggaonuo for induction by Guy11. The results showed that *OsMAPKKK69* transcript accumulation was significantly higher at 12 h post inoculation than in the control treatment, peaking at 36 h post inoculation (*hpi*) (Figure 1B). Although transcript levels began to decline at 48 *hpi*, they remained higher than the control until 72 *hpi*. These results suggest that *OsMAPKKK69* may participate in the rice immune response.

### 2.2. Genetic Characterization of the Osmapkkk69 Mutants

**Figure 2:**
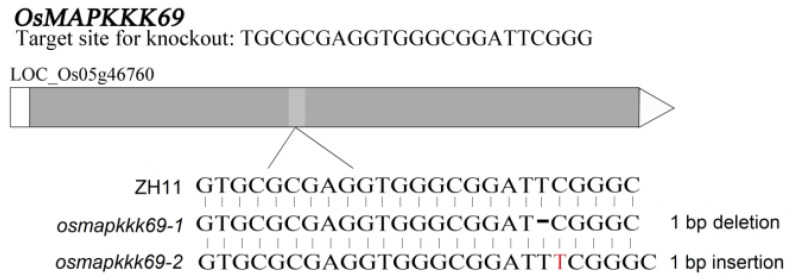
Determination of *osmapkkk69-1* and *osmapkkk69-2* knockout transgenic lines. *Osmapkkk69-1* has 1 bp deletion and *osmapkkk69-2* has 1 bp insertion.

To further determine whether *OsMAPKKK69* is involved in the immune response of rice, we used CRISPR/Cas9 technology to conduct single-target knockout of *OsMAPKKK69* in the ZH11 rice genotype. The 20 nt sequence in *OsMAPKKK69* was selected as the target site for Cas9 cleavage (Supplementary Table S1). After obtaining the knockout mutants, the target sites were sequenced using detection primers (Supplementary Table S1). The results showed that there is a base deletion at the target site for the edited rice line *osmapkkk69-1*, and a base insertion at the target site for the edited rice line *osmapkkk69-2* (Figure 2).

### 2.3. Osmapkkk69 Mutants Are More Resistant to Blast Disease

**Figure 3:**
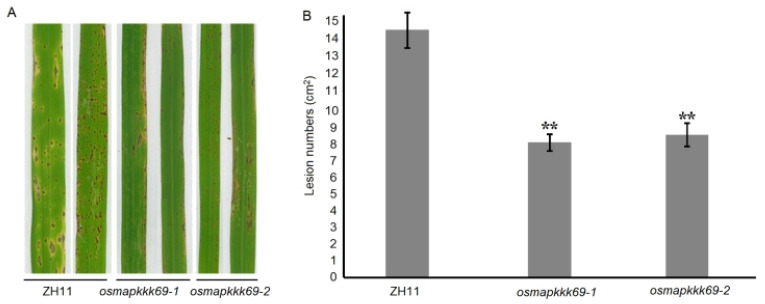
Resistance analysis of the *osmapkkk69* knockout mutants to rice blast caused by the *M. oryzae* Guy11 isolate. (**A**) The plants of *osmapkkk69-1* and *osmapkkk69-2* produced less diseased lesions compared to the ZH11 plants after inoculation with Guy11. (**B**) Lesion numbers per cm^2^ on rice leaves (mean SD, n > 10 leaves) after inoculation with Guy11. The data analysis was conducted using Student’s *t*-test; ** *p* ≤ 0.01.

To further verify the role of *OsMAPKKK69* in the resistance of rice blast in rice, two allelic mutants (*osmapkkk69-1* and *osmapkkk69-2*) and the wild type of ZH11 were inoculated by indoor spray inoculation with the isolates of *M. oryzae* Guy11. The results indicated that the knockout mutants *osmapkkk69-1* and *osmapkkk69-2* were more resistant to rice blast caused by *M. oryzae* Guy11 than that of ZH11 (Figure 3).

### 2.4. Osmapkkk69 Mutants Are Also More Resistant to Bacterial Blight

**Figure 4:**
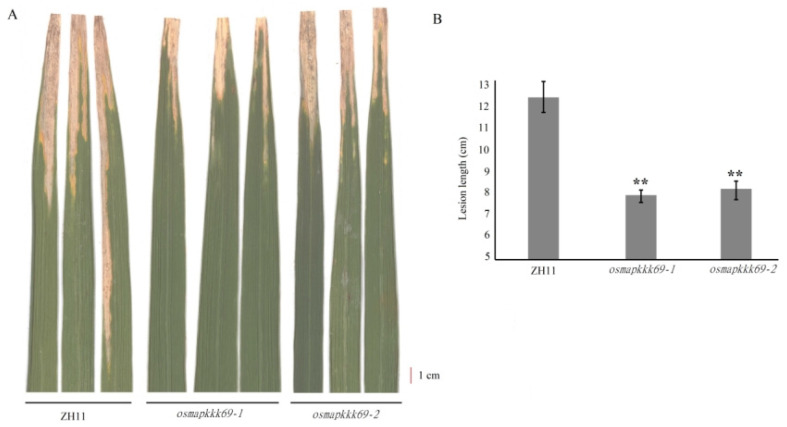
Resistance analysis of *OsMAPKKK69* knockout mutants to bacterial blight. (**A**) *osmapkkk69-1* and *osmapkkk69-2* mutant plants displayed enhanced resistance compared to the ZH11 wild type plants after inoculation with the *Xoo* PXO99 isolate. (**B**) Statistical analysis of the *lesion* length (mean SD, n = 15 leaves) after inoculation with *Xoo* PXO99, conducted using Student’s *t*-test; ** *p* ≤ 0.01 when compared with ZH11.

To assess the effect of *OsMAPKKK69* on the resistance to other pathogens, plants of the abovementioned two allelic mutants (*osmapkkk69-1* and *osmapkkk69-2*) and the ZH11 wild type were inoculated with the *Xoo* PXO99 isolate. The results showed that 14 days after inoculation, the average length of leaf blight in ZH11 plants was 12.23 cm, while the average lengths of leaf blight in *osmapkkk69-1* and *osmapkkk69-2* knockout mutant plants were 7.86 and 8.12 cm, respectively (Figure 4). These results indicate that the *OsMAPKKK69* gene plays an important role in the immune response system of rice and negatively regulates resistance to bacterial blight as well as rice blast.

### 2.5. Analysis of Agronomical Traits of the Osmapkkk69 Mutants and Wild Type ZH11

To further investigate whether *osmapkkk69* mutants affect related agronomical traits, phenotypic comparisons between the *osmapkkk69* mutants and the ZH11 wild type were performed (Table 1). The results showed no significant differences in grain length and seed-setting rate. However, there were significant differences in plant height, panicle length, the number of spikelets per panicle, 1000-grain weight, grain width, and the average yield per plant (Table 1). Further analysis revealed that the grain width of the *osmapkkk69* mutants were smaller, but the grain length and seed-setting rate remained basically unchanged (Supplementary Figure S1).

### 2.6. Temporal and Spatial Transcript Accumulations of *OsMAPKKK69*

**Figure 5:**
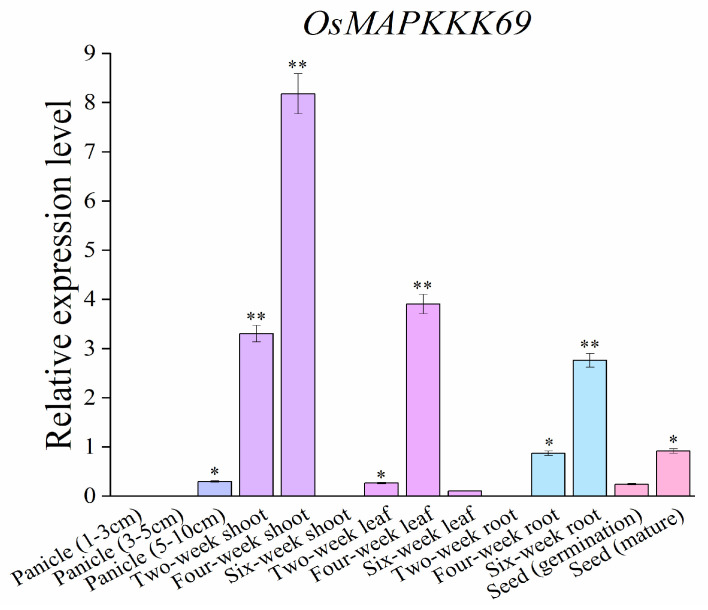
Temporal and spatial expression of *OsMAPKKK69.* The expression levels of *OsMAPKKK69* in shoots, leaves, and roots of 2-, 4-, and 6-week-old seedlings, the panicles of 1–3 cm, 3–5 cm, and 5–10 cm length, as well as germinating and mature seeds were analyzed by RT-qPCR, and the expression levels of *OsMAPKKK69* was highest in four-week shoots. The data analysis was conducted using Student’s *t*-test; * *p* ≤ 0.05, ** *p* ≤ 0.01.

To determine the spatiotemporal expression pattern of *OsMAPKKK69* in different tissues of rice, we extracted RNA from different tissues at different growth stages of plants at 2, 4, and 6 weeks, including a 1–3 cm panicle, 3–5 cm panicle, and 5–10 cm panicle, as well as from mature seeds and germinated seeds. The expression level and profile of *OsMAPKKK69* were analyzed by RT-qPCR (the corresponding primer sequences are shown in Supplementary Table S1). The results indicated that *OsMAPKKK69* was expressed in different tissues of 2-week, 4-week, and 6-week plants, as well as at the panicle and maturity stages, but the expression level of *OsMAPKKK69* was the highest in four-week shoots (Figure 5).

### 2.7. Subcellular Localization of OsMAPKKK69

**Figure 6:**
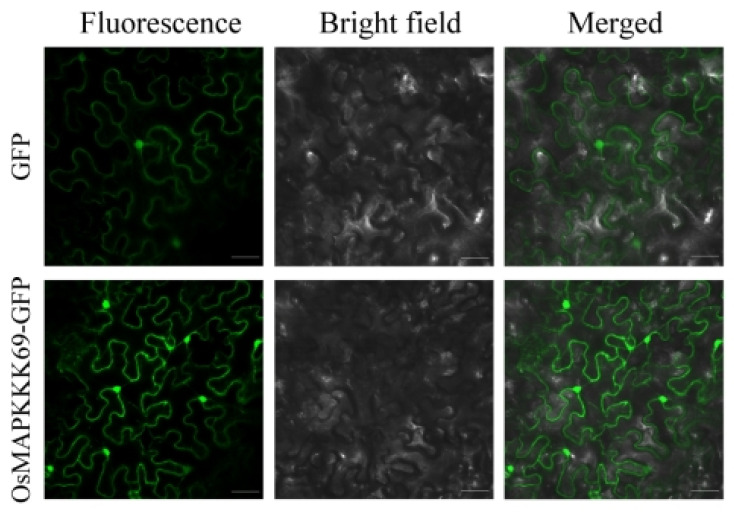
The subcellular localization of *OsMAPKKK69* in leaves of *N. benthamiana*. The OsMAPKKK69 protein is expressed in the cell membrane, cytoplasm, and nucleus. Bar = 20 µm.

*OsMAPKKK69* can negatively regulate the resistance of rice blast, but how does it exert its disease-resistant function? We constructed the 35S: OsMAPKKK69: eGFP vector and transformed it to *Agrobacterium* strain GV3101. Two days after injecting the leaves of *Nicotiana benthamiana* (*N. benthamiana*), we observed the cellular localization of OsMAPKKK69 using laser confocal microscopy technology. The results indicated that OsMAPKKK69 was expressed in the cell membrane, cytoplasm, and nucleus (Figure 6).

### 2.8. Amino Acid Sequence Analysis of OsMAPKKK69 in Different Rice Varieties

**Figure 7:**
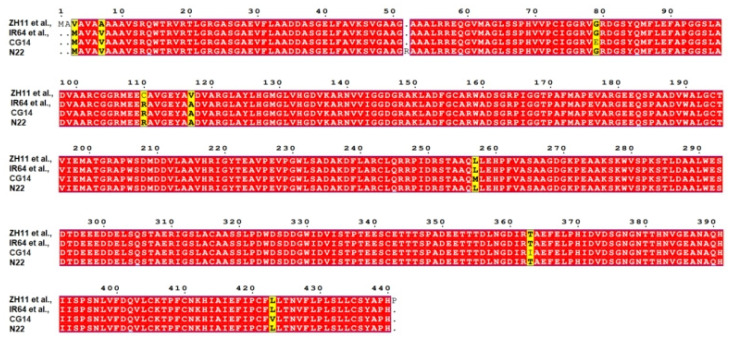
Amino acid sequence analysis of *OsMAPKKK69* in 33 sequenced rice varieties. ZH11, NIP, LJ, KY131, Kosh, and DHX2 showed the same results as *OsMAPKKK69*; IR64, WSSM, Tumba, TM, J4155, III32, G8, FS32, DG, D62, CN1, Basmati1, 9311, Y58S, S548, R527, R498, Lemont, G630, FH838, YX1, Y3551, NamRoo, G46, and 02428 showed the substitution of four amino acids and three amino acids missing; CG14 had eight different amino acid changes and three amino acids missing; N22 has four amino acids replaced, three amino acids missing, and the insertion of one amino acid. Yellow shadings highlight the changed amino acids.

To further analyze the amino acid sequence changes in *OsMAPKKK69* in different rice materials, we found 4 different types of *OsMAPKKK69* changes based on 33 rice varieties that have completed by the 3rd-generation sequencing technologies (https://ricerc.sicau.edu.cn/, accessed on 1 April 2020). The first group of materials comprises the ZH11, NIP, LJ, KY131, Kosh, and DHX2 varieties, and their sequences are exactly the same as that of *OsMAPKKK69*. The second group has the substitution of four amino acids and three missing amino acids in the IR64, WSSM, Tumba, TM, J4155, II32, G8, FS32, DG, D62, CN1, Basmati1, 9311, Y58S, S548, R527, R498, Lemont, G630, FH838, YX1, Y3551, NamRoo, G46, and 02428. The third group, comprising the CG14, has eight different amino acid changes and three amino acids missing. The fourth group, comprising the N22, has four amino acids replaced, three amino acids missing, and the insertion of one amino acid (Figure 7 and Supplementary Table S2). The above results indicate that among the 33 different rice varieties, only 6 rice materials have allele sequences that are the same as the *OsMAPKKK69* sequence of ZH11, while the remaining 27 rice varieties all have different sequences. We speculate that the sequence differences in these alleles are likely to affect the resistance to rice blast and bacterial blight.

## 3. Discussion

### 3.1. OsMAPKKK69 Negatively Regulates the Disease Resistance of Rice

In recent years, studies have shown that the OsMAPKKK family in rice is involved in the plant immune response. Some positively regulate the immune response of rice, such as *OsMAPKKK11*, *OsMAPKKK18*, *OsMAPKKK24*, and *OsMAPKKK67* [13,15,16]. Others negatively regulate the immune response of rice. For example, *OsMAPKKK1* negatively regulates the resistance to bacterial blight [12] and *OsILA1* (*OsMAPKKK43*) negatively regulates the resistance to bacterial blight [14]. However, do other members of the *OsMAPKKK* family also participate in the immune response of plants? This study has discovered, for the first time, that one of the *OsMAPKKK* members, *OsMAPKKK69*, negatively regulates rice blast and bacterial blight in rice.

Studies have shown that the receptor-like cytoplasmic kinase OsRLCK185 interacts with OsMAPKKK11 and OsMAPKKK18, and regulates chitin-induced immune responses through the expression of OsMAPKKK11 and OsMAPKKK18 [16]. Further research has shown that OsRLCK185 transmits immune signals from the PAMP receptor OsCERK1 to the MAPK signal cascade by interacting with the MAPK kinase OsMAPKKK24 and phosphorylating the latter, while OsMAPKKK24 interacts with OsMKK4 and phosphorylates it [15]. Genetic analysis indicated that *OsILA1* (*OsMAPKKK43*) functioned as a negative regulator and acted upstream of the OsMAPKK4-OsMAPK6 cascade in rice–*Xoo* interactions [14]. However, questions remain as to whether *OsMAPKKK69* is involved in regulating chitin-induced immune responses; whether it interacts with receptor-like cell kinases such as OsRLCK185, OsRLCK118, and OsRLCK176; whether it is phosphorylated by these receptor kinases; and whether it can directly phosphorylate OsMAPKK family proteins, etc.

Why did *OsMAPKKK69* act as a negative regulatory factor in this study to regulate the resistance to rice blast and bacterial blight? One explanation, based on the fact that both OsMAPKKK43 [14] and OsMAPKKK69 are localized on the cell membrane (Figure 6), is that OsMAPKKK69 may function in the same way as OsMAPKKK43, by phosphorylating OSMAPKK4 and thereby activating the OSMAPKKK69-OSMAPKK4-OSMAPK6 cascade reaction. Another explanation is that *OsMAPKKK69* may have a similar function to *pi21*. Research shows that *pi21* triggers a slow disease resistance response, and this low-speed induced disease resistance response may be a new mechanism of persistent disease resistance response. The susceptibility allele *Pi21* negatively regulates disease resistance, while the disease resistance allele *pi21* is a loss-of-function mutation [19].

### 3.2. OsMAPKKK69 Simultaneously Negatively Regulates Agronomic Traits Related to the Growth and Development of Rice

Studies have shown that members of the *OsMAPKKK* family in rice not only participate in plant immune responses but also regulate agronomic traits [20]. For example, compared with the wild type, the *OsMKKK10* (*OsMAPKKK10*) mutant had significantly reduced traits such as grain width, grain length, grain weight and spike length [21]; OsMKKK70 has functional redundancy compared with OsMKKK55 and OsMKKK62, which can regulate particle shape [20]. Further studies have shown that compared with the wild type, the seeds of double-sprouted *osmkkkk62/70* and triple-sprouted *osmkkkk55/62/70* were significantly smaller [20].

In this study, it was found that compared with the wild type, the *osmapkkk69* mutants had a smaller grain width (Supplementary Figure S1) and shorter spike length (Table 1) and had a similar phenotype to the *osmkkk10* (*osmapkkk10*) mutants [21]. Based on the phenotypes of *osmkkkk62/70* and triple-sprouted *osmkkkk55/62/70*, we speculate that, compared with the wild type, the seeds of double-sprouted *osmapkkk69/10* were also significantly smaller, and these speculations still require the construction of double mutants for further verification. However, more importantly, this study also found that compared with the wild type, the *osmapkkk69* mutants could affect important traits such as plant height, spike length, effective spike number and even yield (Table 1). The chance that the mutant phenotypes were due to off-target effects of CRIPSR/Cas9 editing is minimal, because the likelihood of both mutants having the same off-target is very low. In conclusion, among the identified rice *OsMAPKKK* gene family, our study is the first to demonstrate that this gene can regulate these agronomic traits.

### 3.3. Application of OsMAPKKK69 in Rice Breeding

The relationship between rice yield and disease resistance is rather complex, as plant diseases consume nutrients and affect growth and yield [3]. On the one hand, when plants are attacked by pathogenic bacteria, they inevitably consume a large amount of nutrient resources to resist diseases, which may affect their growth and development, and even lead to reduced yield [22,23]. On the other hand, enhancing disease resistance without reducing yield has always been an important issue that urgently needs to be solved in the fields of plant pathology and breeding [3,22,23]. For instance, studies have shown that some resistance genes are linked to poor agronomic traits, often at the expense of “sacrificing” yield, making it impossible to achieve both yield and disease resistance [22]. Recent studies have shown that the transcription factor *OsDes1* in rice is a key factor that has a positive regulatory effect on rice yield and disease resistance [23].

This study identified that *OsMAPKKK69* negatively regulated important agronomic traits such as resistance to rice blast, resistance to bacterial blight, tillering, and yield in rice. Most importantly, *osmapkkk69* mutants not only enhanced the resistance to rice blast but also significantly reduced plant height, increased the effective tillering number per plant, and significantly boosted the yield per plant. These agronomic traits are precisely in line with the current breeding goals (Table 1).

Sequence analysis of different genetic materials revealed that *OsMAPKKK69* has a relatively rich variety of variation types (Figure 7 and Supplementary Table S2). Among 33 different rice varieties, 27 varieties had insertions or deletions of 1–3 amino acids (Figure 7). Therefore, in the future, it will be very easy to identify and utilize the excellent alleles or mutant genes of *OsMAPKKK69* in different rice germplasm resources. For example, by detecting the expression level of *OsMAPKKK69* in different germplasm resources, materials with a lower expression level of this gene are screened, and these materials could then be applied to breeding practice.

## 4. Materials and Methods

### 4.1. Plant Materials

The *osmapkkk69-1* and *osmapkkk69-2* mutants generated by the CRISPR/Cas9 approach in the ZH11 rice genotype were used in the current study. A 20 nt sequence in *OsMAPKKK69* was selected as the target site for Cas9 cleavage and generated two different *OsMAPKKK69* knockout mutants designated as *osmapkkk69-1* and *osmapkkk69-2*.

The *osmapkkk69-1* and *osmapkkk69-2* mutant materials were created by Wuhan Aidijing Biotechnology Co., Ltd. (Wuhan, China). The main agronomical rice traits, including, plant height, panicle length, seed-setting rate, 1000-grain weight, grain length, grain width, number of effective panicles, number of spikelets per panicle, and seed setting rate, were investigated and analyzed with reference to Yang et al. [5].

### 4.2. Evaluation of Resistance Between Rice Blast Fungus and Bacterial Blight

The *M. oryzae* isolate Guy11, kindly provided by the State Key Laboratory for Ecological Pest Control of Fujian and Taiwan Crops, Fujian Agriculture and Forestry University, was used in this study. Inoculation of *M. oryzae* Guy11 was performed as previously described by Yang et al. [17]. The filter paper containing Guy11 spores was placed on complete medium and incubated in the dark at 28 °C for one week. Then, it was transferred to rice bran medium and incubated until the mycelia covered the medium. The mycelia were scraped off and spore production was carried out in a 28 °C lighted incubator. After 5 to 7 days, the spores were suspended in water containing 0.2% Tween-20 and the concentration was adjusted to 1 × 10^5^ mL^−1^. The suspension was sprayed evenly onto the surface of rice leaves that had grown for about 15 days. The sprayed seedlings were placed in the dark at 26 °C for 24 h, then placed in a long-day inoculation room with a maintained high humidity environment. After 3 to 5 days, phenotypes were observed, pictures were taken, and the phenotype of the disease was statistically analyzed.

The *Xoo* PXO99 isolate was inoculated, and the grading criteria were applied in accordance with the aforementioned method [24]. The specific measurement method is to measure the length of the disease lesions of each leaf from top to bottom after inoculation. For each mutant, 15 leaves were measured and the average value was calculated.

### 4.3. RT-qPCR Analyses

To further verify the expression of *OsMAPKKK69* induced by pathogenic bacteria, the rice blast fungus Guy11 was used to spray-inoculate the rice variety Shufanggaonuo, which had been grown for about two weeks. Water-treated plants were used as controls. Samples were taken at 0, 12, 24, 36, 48, and 72 *hpi* to quantify the expression level of *OsMAPKKK69* at different time points.

To analyze the expression levels and profile of *OsMAPKKK69,* total RNA was extracted from different plant tissues at different growth stages, including the leaves, shoots, and roots of 2-, 4-, and 6-week-old plants, as well as 1–3 cm panicles, 3–5 cm panicles, and 5–10 cm panicles, in addition to mature seed, germinating seeds, and calli. Total RNA extraction and RT-qPCR analyses were conducted with reference to the methods described by Yang et al. [17]. In addition, *Ubiquitin* was used as the internal reference gene, and the corresponding primer sequences are Ubiquitin-F: AACCAGCTGAGGCCCAAGA and Ubiquitin-R: ACGATTGATTTAACCAGTCCATGA.

### 4.4. Subcellular Localization of OsMAPKKK69

The full-length cDNA of the OsMAPKKK69 gene was amplified and then inserted into the two digestion sites of *EcoRI* and *SmaI* of the pCambia233-35S-EGFP vector to construct the 35S-OsMAPKKK69-eGFP vector. Then, the constructed vector was transformed into *Agrobacterium* strain GV3101 and injected into *N. benthamiana* leaves. Three days after injection, the GFP signal was observed and imaged using a Zeiss LSM 880 confocal microscope (Carl Zeiss AG, Oberkochen, Germany).

### 4.5. Bioinformatics Analysis

Multiple sequence alignments are conducted with reference to the following database: http://multalin.toulouse.inra.fr/multalin/multalin.html (accessed on 1 July 2025).

## Figures and Tables

**Table 1:** Comparison of the main agronomic traits between *OsMAPKKK69* knockout lines and ZH11.

Traits	ZH11	*Osmapkkk69-1*	*Osmapkkk69-2*
Plant height (cm)	119.75 ± 2.82	78.25 ± 2.73 **	79.27 ± 2.67 **
Panicle length (cm)	23.42 ± 0.80	20.41 ± 0.52 *	20.83 ± 0.62 *
Number of effective panicles	9.75 ± 0.63	14.01 ± 1.08 **	14.34 ± 1.10 **
Spikelets per panicle	166.15 ± 5.12	148.32 ± 4.15 *	146.24 ± 4.22 *
Seed-setting rate (%)	86.39 ± 1.84	85.62 ± 4.19	86.12 ± 3.68
1000-grain weight (g)	27.27 ± 1.38	25.15 ± 1.80 *	24.59 ± 1.45 *
Grain length (mm)	7.44 ± 0.04	7.41 ± 0.08	7.43 ± 0.06
Grain width (mm)	3.34 ± 0.06	3.16 ± 0.08 *	3.15 ± 0.07 *
Average yield per plant (g)	38.16 ± 0.92	44.94 ± 1.13 *	44.62 ± 1.10 **

Note: the data analysis was conducted using Student’s *t*-test; * *p* ≤ 0.05 and ** *p* ≤ 0.01 compared to ZH11.

## Data Availability

Data are contained within the article and Supplementary Materials.

## Supplementary Materials

The following supporting information can be downloaded at: https://www.mdpi.com/article/10.3390/plants14162566/s1, Figure S1: Comparison of rice grain shapes between the *osmapkkk69* mutants and the wild type ZH11; Table S1: Primer sequences used for synthesizing gRNA spacers and genotyping CRISPR-edited mutants; Table S2: The changes of the OsMAPKKK69 gene in 33 rice varieties.
