# Supplementary material for: OsMAPKKK69 Negatively Regulates Resistance to Blast and Bacterial Blight Diseases in Rice (Oryza sativa L.)
Source: Plants (Basel). 2025 Aug 18;14(16):2566. doi: 10.3390/plants14162566 (PMC12389254; doi:10.3390/plants14162566)
Supplement: Supplementary file 1 [file plants-14-02566-s001.zip › Supplementary Table S1.pdf]

Supplementary Table S1. Primer sequences used for synthesizing gRNA spacers and genotyping CRISPR-edited mutants

Name	sequence 5'→3'	Purpose
gRNAs-*OsMAPKKK69*	TGCGCGAGGTGGGCGGATTCGGG	CRISPR/CAS9
OsMAPKKK69-F	AAGGCGAGGAATGTCGTGAT	Screening of lines
OsMAPKKK69-R	GTCCATGTCGCTCCACGG	Screening of lines
OsMAPKKK69-cDS-F	GACAAAGAGGGAACCAACAG	Gene amplification
OsMAPKKK69-cDS-R	CTAAGGGTGAGGAGCGTATG	Gene amplification
OsMAPKKK69-qRT-PCR-F	TGGAGCGACATGGACGACG	qRT-qPCR
OsMAPKKK69-qRT-PCR-R	GGCGACGAACGGGTGTTCTA	qRT-qPCR
OsMAPKKK69-1300GFP-F	CTGCAGGGGCCCGGGGTCGACATGGCGGTGGC GGTGGCGGC	Cell localization
OsMAPKKK69-1300GFP-R	CCCTTGCTCACCATGGTACCAGGGTGAGGAGC GTATGAAC	Cell localization
UBIQUITIN-F	AACCAGCTGAGGCCCAAGA	Reference gene
UBIQUITIN-R	ACGATTGATTTAACCAGTCCATGA	Reference gene
