# Supplementary figures and images for: OsMAPKKK69 Negatively Regulates Resistance to Blast and Bacterial Blight Diseases in Rice (Oryza sativa L.)
Source: Plants (Basel). 2025 Aug 18;14(16):2566. doi: 10.3390/plants14162566 (PMC12389254; doi:10.3390/plants14162566)

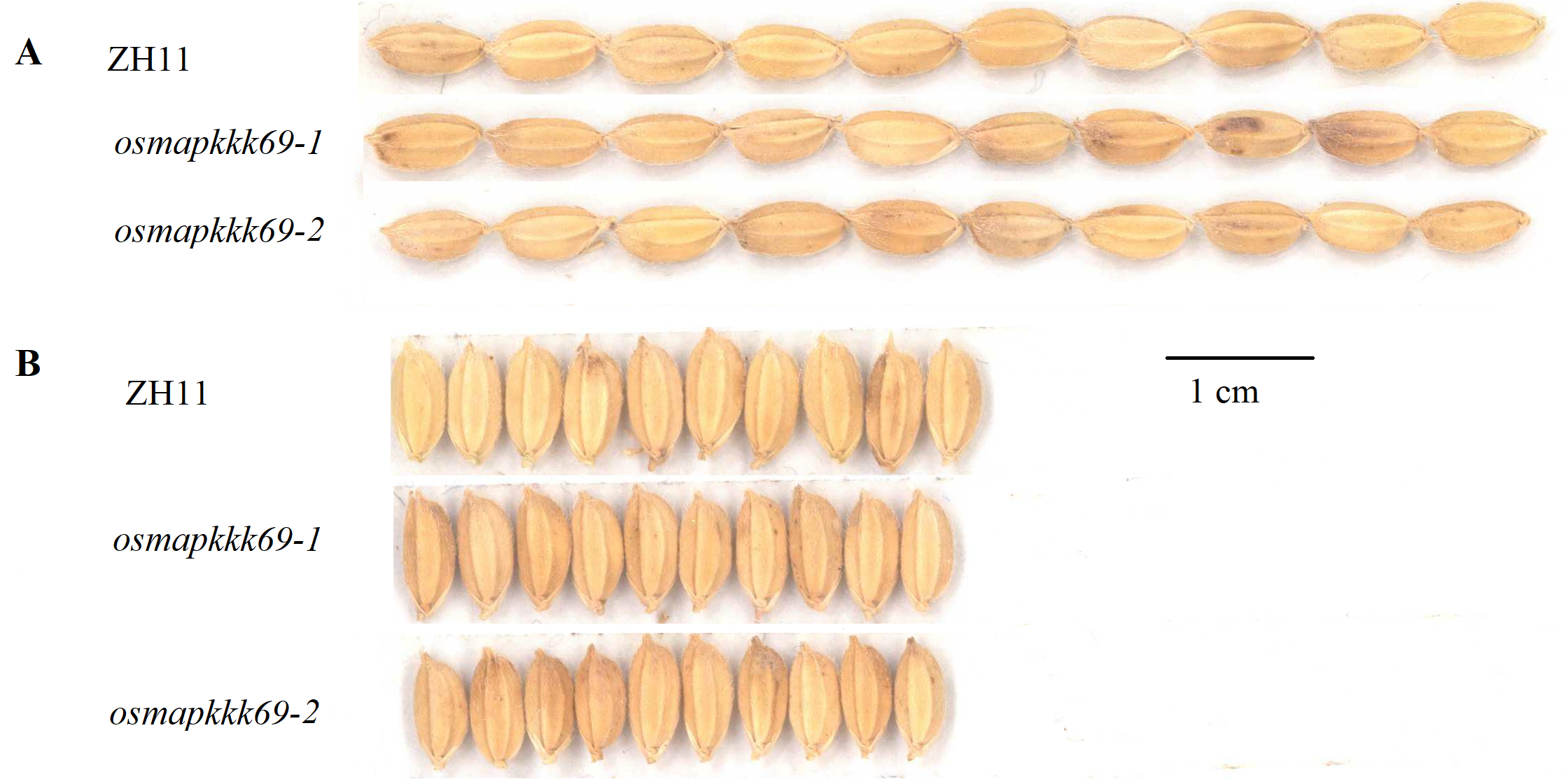

Supplement: Supplementary file 1 [file plants-14-02566-s001.zip › Supplementary Figure S1.png]
